# Catheter-Electrode System for Continuous Aptamer-Based Sensing in the Rat Subcutaneous Space

**DOI:** 10.1149/2754-2726/ae048c

**Published:** 2025-09-19

**Authors:** John Mack, Yao Wu, Yuchan Yuan, Remy Bell, Netzahualcóyotl Arroyo-Currás

**Affiliations:** 1 Biochemistry, Cellular and Molecular Biology Program, Johns Hopkins School of Medicine, Baltimore, Maryland 21205, United States of America; 2 Department of Pharmacology and Molecular Sciences, Johns Hopkins School of Medicine, Baltimore, Maryland 21205, United States of America; 3 Department of Biomedical Engineering, University of South Carolina, Columbia, South Carolina 29208, United States of America

**Keywords:** aptamer, in vivo, stereolithography, in-vivo biosensing, pharmacology, electrochemical sensors

## Abstract

Electrochemical aptamer-based (E-AB) sensors can continuously monitor drug concentrations with high temporal resolution. While previous studies have successfully demonstrated in vivo molecular monitoring using E-AB sensors, they typically rely on custom-built probes fabricated through manual, labor-intensive processes. Constructing such probes requires substantial time, technical expertise, and precision, creating a high barrier to entry for researchers interested in the field of in vivo measurements. To address this limitation, we present an alternative fabrication approach that combines established device construction methods with rapid prototyping via additive manufacturing (i.e., stereolithography). This platform enables continuous molecular monitoring in the subcutaneous interstitial fluid (ISF) of rodent models. We selected ISF as a target fluid due to its growing recognition as a promising frontier for continuous molecular monitoring and its central role in current biosensor research. Our goal is to eliminate the most prohibitive steps in traditional fabrication, thereby reducing the skill threshold required for building functional E-AB probes. By improving the construction process, our methods should make the technology more easily adoptable by a broader range of research laboratories. Ultimately, we aim to broaden the use of E-AB sensors and accelerate development in the field of real-time, in vivo molecular monitoring.

## Highlights

- We report an electrode probe/catheter system that is simple to construct via 3D printing technology and commercially available materials.
- This system supports reproducible electrochemical aptamer-based (E-AB) sensors using three different aptamer sequences.
- The catheter can be implanted into the subcutaneous space of anaesthetized rodents with minimal surgery, and the probe securely locked into place.
- Proof-of-concept in vivo measurements show that a probe modified with an E-AB vancomycin sensor is capable of measuring the diffusion of vancomycin into the interstitial fluid of the subcutaneous space following IV dosing.

## Graphical abstract

**Figure:**
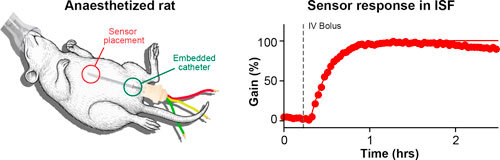

Continuous measurement technologies for real-time monitoring of the human body are becoming essential for advancing preventive medicine.^1,2^ By enabling individuals to continuously monitor biochemical changes in their body during daily activities, these tools would allow early detection of disease pathology and empower prophylactic intervention preceding symptom onset. The clinical and commercial success of enzyme-based continuous glucose monitors (CGMs) highlights the potential of this approach. CGMs have transformed diabetes management by eliminating the need for frequent finger-prick tests and providing high-resolution glucose data to support timely medical interventions. This technology has evolved into sophisticated closed-loop systems, such as artificial pancreas devices, that integrate real-time glucose sensing with automated insulin delivery feedback.^3^ The success of CGMs suggests that similar platforms for other target molecules could significantly enhance care for a variety of conditions rooted in molecular imbalances. However, the enzymatic mechanisms leveraged by CGMs; specifically, their dependence on a particular redox process, poses a major barrier to adapting this approach for other analytical targets. As a result, researchers have shifted focus to alternative strategies—particularly affinity-based sensing technologies—to enable continuous, real-time molecular surveillance of relevant molecules beyond glucose.

One such promising technology that has gained traction in recent years is the aptamer-based sensor.^4,5^ This platform uses short oligonucleotide sequences—known as aptamers—selected through systematic evolution of ligands by exponential enrichment (SELEX) for high affinity and specificity toward target molecules. Aptamers can be engineered to undergo structural changes upon target binding, forming the basis for signal transduction. Aptamer sensors offer several advantages for in vivo molecular monitoring. They can be synthesized cost-effectively and reproducibly via well-established solid-phase methods,^6^ and their selection process can be tailored to recognize a vast range of molecular targets.^7^ Furthermore, small molecule binding is reversible with rapid association and dissociation rates on the millisecond scale, supporting real-time tracking of dynamic increases and decreases in target concentration.^8^ A notable implementation of this technology is the electrochemical aptamer-based (E-AB) sensor,^9^ which leverages the structure-switching behavior of aptamers in conjunction with a covalently linked redox reporter to generate measurable electrochemical signals that reflect the kinetics of target binding.^9,10^ Recent advances demonstrate that these sensors can achieve continuous molecular monitoring for clinically relevant targets in living subjects across various body compartments.^11–13^ Furthermore, E-AB sensors present a unique and powerful platform for investigating site-specific pharmacokinetics of therapeutic agents in spaces such as tumor microenvironments.^14^

Despite the promise of current in vivo E-AB sensors, complexity in both device fabrication and surgical implantation limits broader adoption, particularly among research groups lacking specialized expertise. To address this barrier, we have developed a streamlined platform designed to simplify both sensor production and in vivo deployment in the subcutaneous space (i.e., analogous to CGMs). In this study, we present a proof-of-concept device that leverages stereolithographic 3D printing and readily available materials to create E-AB sensors that can support continuous monitoring of a model analyte in anesthetized rodents. The device features a bundled wire electrode array (Figs. 1A, 1B) comprising a gold working electrode, platinum counter electrode, and silver pseudo-reference electrode that interlocks with a hollow catheter (Fig. 1C), facilitating minimally invasive subcutaneous implantation (Fig. 1D). This approach aims to provide a cost-effective (∼$6 USD for components per device, approximately one hour of active construction time to create a fully assembled electrodes-in-catheter probe) and accessible test bed for in vivo evaluation of E-AB sensors. By lowering the technical barriers to entry, we hope to expand the experimental capabilities of research groups working to advance continuous molecular sensing.

**Figure 1.**
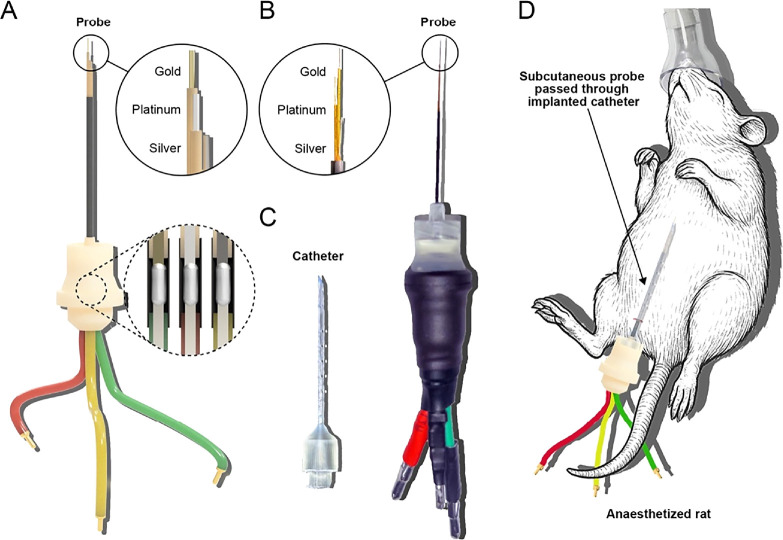
3D-printed, aptamer-based electrochemical probe for continuous molecular monitoring in the subcutaneous space. (A) Rendered image of the electrode probe designed in Fusion 360 software. *Solid line inset*: rendering of the probe tip, displaying the components of the three-electrode cell. *Dashed line inset:* rendering of soldered connections between the working, reference, and counter electrode wires and the rear-mounted connectors compatible with potentiostat clamps. (B) Bright-field image of the fully assembled electrode probe. *Solid line inset:* image of the staggered electrochemical cell, corresponding to the solid line inset from A. (C) Photograph of the custom 3D-printed implantation catheter. (D) Overview of the proposed approach for continuous in vivo sensing, illustrating probe and catheter placement in the subcutaneous space of an anesthetized rodent.

## Materials and Methods

### Reagents

5’ thiol and 3’ methylene blue-modified oligonucleotides were purchased from Sigma Aldrich (Houston, TX) and diluted in 1 × Tris-EDTA (Sigma Aldrich PN: 93283) to a stock concentration of 100 μM. Sodium hydroxide (Fisher Scientific PN: S318), sulfuric acid (Fisher Scientific PN: A510), and tris(2-carboxyethyl)-phosphine hydrochloride (Sigma Aldrich PN: C4706) were diluted to working concentration in water. 6-mercapto-1-hexanol (Sigma Aldrich PN: 451088), oligonucleotide stock, vancomycin hydrochloride (ThermoFisher Scientific PN: J62790), and tobramycin sulfate (GoldBio, PN: T-341–1) were diluted in phosphate-buffered saline (PBS, 11.9 mM HPO3^2−^; 137 mM NaCl; 2.7 mM KCl; pH = 7.4) with 2 mM magnesium chloride (Fisher Scientific PN: BP214). Any water used was purified by a Mili-Q Direct system with resistivity of 18 MΩ.

### Stereolithographic (SLA) 3D printing of components

The catheter and probe shell (Fig. S1) were fabricated using a FormLabs 3B+ SLA printer (FormLabs, Boston MA) with Clear Resin V4.1 (FormLabs, Boston MA). After printing, all components were washed in 100% isopropanol (Sigma Aldrich, PN: 278475) for 15 min in a washing chamber. The catheter bore and probe shell channels were then manually flushed with isopropanol using a syringe. Printed parts were air-dried overnight in a cool, dark environment, followed by UV curing at 50 °C for 15 min (*λ* = 405 nm) in a curing chamber.

### Probe assembly

Six-centimeter segments of 0.05 mm-diameter gold (ThermoScientific, PN: 010969), platinum (ThermoScientific, PN: 013479), and silver (ThermoScientific, PN: 044461) wire were each threaded through hollow fused silica capillaries (Polymicro Technologies, PN: 1068150018) with an inner diameter of 0.0743 mm and lengths of 5 cm, 4.7 cm, and 4.5 cm, respectively. For each wire, approximately 2 mm was left exposed at one end, with the remaining length (difference between six-centimeter segments and corresponding capillary length) protruding from the opposite end. Both ends of each capillary were sealed with a droplet of bonding resin (Let’s Resin, Amazon), to prevent liquid leakage into the capillary. The longer exposed end of each wire was soldered to a breakout wire, and the solder joints were insulated using heat-shrink tubing. The wires were then inserted into the three separate channels of the 3D-printed shell (Fig. S1C), positioning the soldered ends to ensure the 2 mm exposed segments were staggered and not in contact. The assembly was sealed with bonding resin and inserted through the Luer end of the probe shell (Fig. S1D). The internal cavity was filled with epoxy resin for reinforcement and sealing, and additional heat-shrink tubing was applied to secure the breakout wires and adhesion points. Finally, a length of PTFE tubing (1 mm inner diameter) was used to reinforce the bundle of fused silica capillaries on the probe side, leaving the 2 mm wire segments exposed.

### Electrode and E-AB sensor preparation

To prepare the gold wire electrode for aptamer deposition, the probe assembly was first immersed in 0.5 M sodium hydroxide and the gold wire was subjected to cyclic voltammetry (CV) between –1.0 V and –1.8 V vs a pseudo reference Ag wire for 500 cycles at a potential scanning rate of 1 V s^−1^. The probe assembly was then transferred to 0.5 M sulfuric acid (H_2_SO_4_) and scanned from 0.2 V to 1.6 V for 120 cycles at the same scan rate. To homogenize electrode to electrode surface areas and increase aptamer loading, we electrochemically roughen the gold surface via chronoamperometric pulsing between 0 V and 1.9 V for 16,000 cycles, with a 10 ms pulse width and 1 ms sample interval. The cell was intermittently tapped during pulsing to dislodge gas bubbles that accumulated on the electrode surface. The roughened electrode surface area was then characterized via cyclic voltammetry in 0.05 M H_2_SO_4_, scanning from 0.2 V to 1.6 V for two cycles. Immediately after the final CV cycle, the probe was immersed in an aptamer solution. Prior to electrode incubation, the aptamer had been reduced in 50 mM tris(2-carboxyethyl)phosphine (TCEP) for one hour in the dark and diluted to the working concentration indicated. The probe was incubated in the aptamer solution for one hour at room temperature, then transferred to a solution 3 mM in mercaptohexanol (MCH) for monolayer backfilling. The probe assembly was incubated in MCH for 12–18 h at 25 °C, then transferred to phosphate-buffered saline (PBS) and stored at 4 °C if not used immediately. All electrochemical procedures were conducted using a CH Instruments Model 1040 C electrochemical analyzer (CH Instruments, Austin, TX).

### Sensor calibration

Calibration curves were constructed by serially adding target analyte to phosphate-buffered saline (PBS) containing 2 mM magnesium, during continuous interrogation via square wave voltammetry (SWV). SWV was performed using an amplitude of 25 mV at aptamer-specific “signal ON” and “signal OFF” frequencies. Each E-AB sensor was scanned at both frequencies in sequence, approximately once per minute. Target concentrations were increased logarithmically, with additions made following every 11th voltammetric scan (∼10 min apart). Only the voltammogram from the 11th scan at each concentration step was used for further analysis. For each concentration point, the change in SWV peak height relative to the 0 μM target baseline (i.e., signal gain) was plotted as a function of target concentration for both ON and OFF frequencies. These data were used to calculate the kinetic differential measurement (KDM), which was then plotted against the logarithm of target concentration and fit to the Hill equation as follows:

\begin{eqnarray*}{\mathrm{Gain}}\left({\mathrm{target}}\right)={\mathrm{base}}+\left\{\left({\mathrm{\max }}\,-{\mathrm{base}}\right)/\left(1+{\left({{\mathrm{EC}}}_{50}/\left[{\mathrm{target}}\right]\right)}^{{\mathrm{rate}}}\right)\right\}\end{eqnarray*}

The numerical constants *base*, max, *EC*_50_, and *rate* were calculated by nonlinear regression and represent the minimum gain, gain at sensor saturation, concentration at half of the maximum gain, and the Hill coefficient, respectively. The concentration of target was designated the independent variable and represented as *target*.

### In vivo measurement

In vivo experiments were conducted on male Sprague-Dawley rats weighing between 300–400 g. The rat was induced with 5% isoflurane in oxygen for 10 min before the surgery and maintained at 2% throughout the procedure. All animal procedures were approved by the Johns Hopkins Animal Care and Use Committee under protocol RA22M242 and were conducted in accordance with the Guide for the Care and Use of Laboratory Animals. After ensuring that the rat was fully under anesthesia, the rat’s abdominal area was shaved, and a small (∼1 mm) incision was made in the dermis using surgical scissors. A catheter was gently inserted into the subcutaneous space, taking care to avoid penetrating deeper tissues or causing bleeding. The catheter was then filled with phosphate-buffered saline (PBS) using a syringe, and the probe was inserted and secured using the Luer lock mechanism. The catheter assembly was affixed with surgical tape, and the incision site was covered with damp gauze to prevent tissue drying. Once the circuit was confirmed to be intact, the E-AB sensor was continuously interrogated using square wave voltammetry (SWV). Vancomycin was administered intravenously via the tail vein at the indicated time point. The kinetic differential measurement (KDM) signal gain was plotted as a function of time and fit to the following equation. The fitting was restricted to the portion of the curve between the maximum and minimum post-injection signal gain values:

\begin{eqnarray*}{\mathrm{Gain}}\left({\mathrm{t}}\right)={{\mathrm{G}}}_{{\mathrm{\max }}}-{{\mathrm{Ae}}}^{-{\mathrm{kt}}}\end{eqnarray*}

Where *G*_max_, *k*, and *A*, are constants representing the signal gain plateau, equilibrium time constant of target diffusing into the subcutaneous space, and a correction factor to account for delayed signal increase, respectively, and *t* is time.

### Data analyses

All square wave voltammetry (SWV) data were analyzed using the open-source Python software package SACMES,^15^ previously developed and published by our laboratory. Data fitting and graphical representations were performed using Igor Pro v8.0 (WaveMetrics, Lake Oswego, OR). Calibration experiments were conducted with *n* = 5, while in vivo measurements were performed with *n* = 1. Shaded regions in all plots represent the standard deviation.

## Results and Discussion

The goal of this project was to create an alternative E-AB probe design that could be (1) manufactured via stereolithography (SLA) using a standard (<$4,000 USD) benchtop SLA printer and (2) implanted subcutaneously to allow in vivo molecular monitoring in an anesthetized rodent model. We based our design on the original in vivo E-AB probe,^16–18^ which consisted of three microwires independently insulated with shrink tubing and bundled together to form the electrode probe. This original design presented two key challenges: First, the segment of wire exposed to the in vivo environment required manual stripping of insulation to enable aptamer-based monolayer functionalization, a process demanding significant manual skill and carrying the risk of damaging the wire and wasting materials. Second, the bundled wires were fragile and highly flexible, complicating stable electrical connections with conventional alligator clips used in commercial potentiostats. Movement during setup or subsequent procedures often led to wire breakage and irreversible probe loss.

We acknowledge that more recent studies, such as Leung et al.^19^ have addressed the first issue by using pre-uninsulated wires, thereby eliminating the need for manual insulation removal. However, these designs still rely on exposed wires for back-end connections, which remain susceptible to mechanical failure during repeated handling. In contrast, our approach integrates the staggered geometry of the original in vivo probe with robust electrical insulation and connection strategies inspired by fast-scan cyclic voltammetry (FSCV) probes,^20^ improving both durability and ease of use.

**Figure 2.**
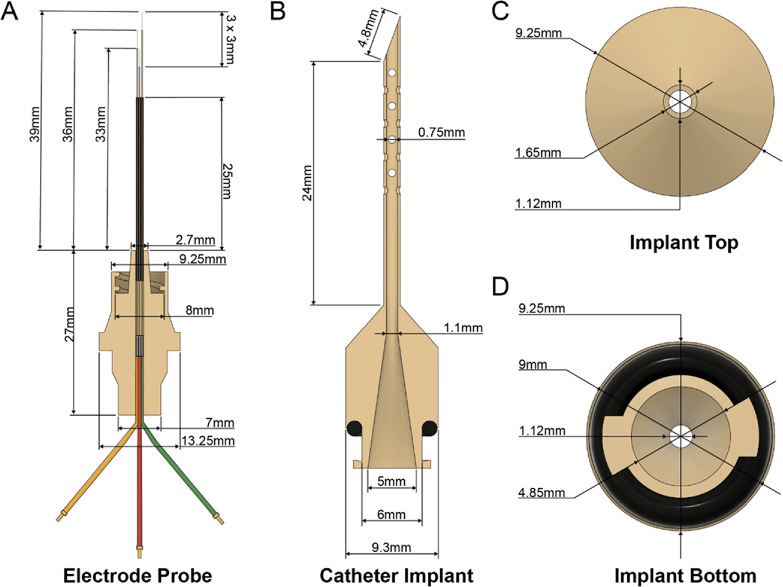
Design of probe and catheter assembly. (A) The probe includes three staggered wired electrodes—working (gold), counter (platinum), and pseudo-reference (Ag/AgCl-coated silver)—each made from 50 μm-diameter wires. The electrodes are encased in fused silica capillaries for structural support and connected to contact wires via soldering at a stereolithographically printed base. (B) The catheter is a single, stereolithographically printed piece through which the probe is inserted. (C) Its dimensions and taper resemble those of a 20 G medical catheter. (D) The inner chamber is smooth, allowing frictionless sliding of the probe to prevent sensor damage.

Our alternative probe assembly integrates a three-electrode electrochemical cell with a printed scaffold containing a built-in male Luer lock (Fig. 2A). This matches to a corresponding female fitting at the base of a perforated smoothbore catheter (Fig. 2B), designed to be rigid enough to penetrate the skin and act as protective cage for the electrodes. Furthermore, the catheter contains holes that allow entry of the interstitial fluid (ISF) into the device and fully wet the E-AB probe. The catheter top is smooth (Fig. 2C) to allow frictionless sliding into the subcutaneous compartment. For a more in-depth description of device construction, please see the **Methods**.

The catheter shown in Fig. 2 can be easily implanted into the subcutaneous space of an anaesthetized rat without the need for major surgery by first making an approximately 1 mm-wide incision in the desired implantation area and manually pressing the catheter through the hole and under the skin. This presents a significant simplification from previous works that require surgical exposure and dissection of the jugular vein, followed by re-suturing.^17^ Furthermore, it presents an alternative to microneedles for measuring drug concentration dynamics in ISF.^13^ It can then be loaded with a small amount of physiological saline to hydrate the wound and flush any dermal materials lodged into its bore. Because the base of the catheter has a smooth bore (Fig. 1D), the E-AB probe can be easily fed through after catheter implantation without damage and locked into place via the Luer lock. This creates a watertight seal that prevents the probe from drying and firmly anchors the setup in place to reduce noise caused by physical disturbance of the probe.

**Figure 3.**
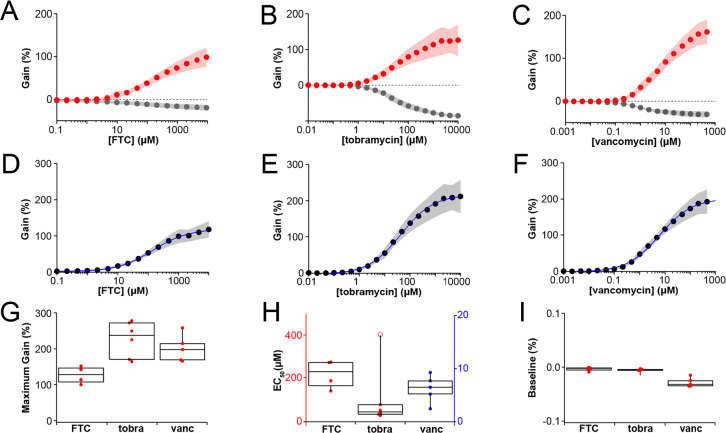
Dose-response and thermodynamic analysis of sensors functionalized with three different aptamers. (A) FTC aptamer (F_on_ = 600 Hz, F_off_ = 8 Hz), (B) tobramycin aptamer (F_on_ = 300 Hz, F_off_ = 30 Hz), and (C) vancomycin aptamer (F_on_ = 250 Hz, F_off_ = 10 Hz) sensors were challenged with increasing concentrations of their respective targets. Dose-response curves were generated using dual-frequency square wave voltammetry (SWV) and (D)–(F) kinetic differential measurements (KDM). Red points indicate signal at high (“ON”) frequency; grey points indicate low (“OFF”) frequency; black points indicate percent gain from KDM; blue lines in KDM traces show nonlinear fits to the Hill equation. For each aptamer, *n* = 5 sensors; shaded regions represent ± one standard deviation. (G)–(I) Thermodynamic parameters extracted from the fitted curves: (G) maximum gain, (H) EC_50_, and (I) baseline gain. Box plots display interquartile ranges (boxes), medians (center lines), and full data range (whiskers). Statistical outliers are represented by open circles and were excluded from calculations.

Using the probe design shown in Fig. 2, we evaluated the baseline performance of our electrodes functionalized with three different aptamers to demonstrate the platform’s broad sensor applicability. As proof of concept, we tested sensors in vitro (Fig. 3) using aptamers targeting three distinct drug classes: the aminoglycoside antibiotic tobramycin, the glycopeptide antibiotic vancomycin,^21^ and the reverse transcriptase inhibitor emtricitabine (FTC).^22^

To fabricate E-AB sensors, each aptamer was immobilized onto a gold wire electrode via a thiol monolayer. Prior to aptamer deposition, the electrode underwent a multi-step electrochemical treatment to prepare the surface.^23^ First, organic contaminants were removed through voltammetric cycling in aqueous sodium hydroxide solution at negative potentials. The surface was then activated by scanning in aqueous sulfuric acid solution across the gold potential window. Finally, the electrode was roughened via potential pulsing to enhance aptamer loading.^24^ A final scan in 0.05 M sulfuric acid at a low scan rate was used to estimate the electrochemically active surface area (Figs. S2A–S2B). Following surface preparation, electrodes were incubated in aptamer solution for one hour and subsequently backfilled with mercaptohexanol overnight to complete monolayer formation. Aptamer deposition concentrations used are 500 nM for FTC and vancomycin aptamers, and 1 μM for the tobramycin aptamer. The assembled sensors were then challenged with serially increasing concentrations of target analyte to generate calibration curves. Specific details regarding these procedures are described in detail in the **Methods**.

Square wave voltammetry (SWV) was employed to interrogate the sensors, as it offers a high signal-to-noise ratio. To mitigate sensor to sensor variability and signal drift while enhancing analytical performance, both high (“ON”) and low (“OFF”) square wave frequencies were used, selected based on prior published optimization data to maximize concentration-dependent signal changes.^21,22,25^ Sensors were continuously scanned in PBS while the target molecule concentration was progressively increased. Resulting SWV peak heights were normalized to the signal obtained at [target] = 0 mM and plotted against concentration (Figs. 3A–3C). To correct for sensor-to-sensor variability in fabrication and further enhance sensitivity, normalized signals were used to calculate a kinetic differential measurement (KDM),^15^ the equation for which is as follows:

\begin{eqnarray*}\begin{array}{c} \% \,{signal}\,{change}\,\left({\left[{target}\right]}_{n}\right)\\ =\,\left\{\frac{{i}_{p,{ON}}\left({\left[{target}\right]}_{n}\right)}{{i}_{p,{ON}}\left({\left[{target}\right]}_{0}\right)}-\frac{{i}_{p,{OFF}}({\left[{target}\right]}_{n})}{{i}_{p,{OFF}}({\left[{target}\right]}_{0})}\right\}\times 100\end{array}\end{eqnarray*}

These KDM values were then plotted against concentration and fit to the Hill equation via nonlinear regression to produce calibration curves (Figs. 3D–3F). From these fits, we extracted key performance metrics: maximum gain (Fig. 3G), EC_50_ (Fig. 3H), and baseline signal (Fig. 3I), which were statistically compared across sensors.

Overall, the calibration curves shown in Fig. 3 were consistent across sensors functionalized with the same aptamer and covered a broad concentration range suitable for in vivo applications. Except for a single outlier, thermodynamic parameters (gain, EC_50_, baseline) showed minimal variability, demonstrating strong reproducibility and low sensor-to-sensor variation. Analyses pertaining to surface density for each sensor are included in Figs. S2C–S2D.

**Figure 4.**
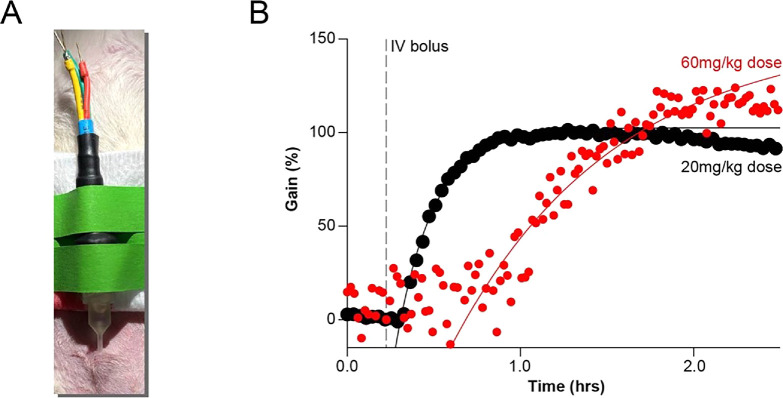
Continuous E-AB monitoring of vancomycin uptake from blood into the subcutaneous space. (A) Photograph of one rat with electrode-catheter assembly implanted in the abdominal subcutaneous space. (B) Signal gain over time for sensors implanted in two anesthetized rats, continuously interrogated using dual-frequency SWV. The dashed gray line indicates the time of IV bolus administration of vancomycin at either 20 mg kg^−1^ (black trace) or 60 mg kg^−1^ (red trace). Solid lines represent nonlinear regression fits to a mono-exponential model describing diffusion of vancomycin from the bloodstream into the subcutaneous space.

To demonstrate the proof-of-concept in vivo deployment of our probe-catheter system, we conducted preliminary testing in Sprague-Dawley rats. As this study served as an initial feasibility assessment, our approved protocol required the use of euthanasia-designated animals that had previously undergone other surgical procedures. For sensor measurements, rats were anesthetized with isoflurane according to the protocol detailed in the **Methods**. A small (∼1 mm) superficial incision was made in the abdominal skin to facilitate catheter implantation. The catheter was carefully inserted between the skin and the panniculus carnosus, ensuring that the insertion path remained parallel to the subcutaneous plane and did not penetrate underlying muscle tissue. To aid placement and minimize tissue disruption, the catheter was moistened with PBS prior to insertion. A vancomycin-specific E-AB sensor was then introduced into the catheter, secured in place (Fig. 4A), and continuously interrogated using dual-frequency SWV. After collecting a 15 min baseline, vancomycin was administered via a tail vein intravenous bolus, and data collection continued for 2 h to monitor drug absorption from the bloodstream into the rat’s ISF. The resulting KDM sensorgrams (Fig. 4B) revealed a drug uptake profile that plateaued within the observation window. Data were fit to a mono-exponential growth model using nonlinear regression, as described in the **Methods**. This analysis yielded uptake rate constants of 4.55 ± 0.18 h^−1^ for a 20 mg kg^−1^ dose and 1.06 ± 0.18 h^−1^ for a 60 mg kg^−1^ dose, the latter of which is in good agreement with previously reported values for vancomycin diffusion in subcutaneous tissues.^26^

We tested two doses (20 mg kg^−1^ and 60 mg kg^−1^) in two independent animals. The higher dose produced a greater signal plateau, indicating a higher local drug concentration, but also showed a delayed signal onset. This delay is likely attributable to physiological variability in circulation status, as the animals had been previously subjected to unrelated surgical procedures. Additionally, greater signal scatter was observed in the high-dose data, which we attribute to noise in the measurement environment. Given these constraints and the ethical consideration to limit animal use, we chose not to pursue further testing within this preliminary study. However, we are currently preparing revised animal protocols to support expanded, rigorously controlled studies using dedicated experimental groups. Despite these limitations, the data presented in Fig. 4 clearly demonstrate successful in vivo deployment of our catheter-probe system and real-time molecular monitoring of vancomycin uptake from blood into the ISF in rats.

## Conclusions

In this study, we present a simplified method for continuous in vivo molecular sensing in the subcutaneous space of anesthetized rats. This approach offers an alternative to previously reported in-vein sensing platforms,^12,17,18^ with several notable advantages: it uses accessible materials, involves straightforward fabrication and assembly, offers improved durability, exhibits low device-to-device variability, and allows for implantation without the need for complex surgical procedures. Importantly, this sensing paradigm is highly adaptable. By modifying the 3D-printed probe housing, it could be extended to various form factors and experimental setups. We also demonstrate that E-AB sensors integrated into this platform can track changes in drug concentration within the subcutaneous space following intravenous drug administration.

The catheter approach presented in this study narrowly focuses on demonstrating the design, construction and functionality of 3D printed scaffolds to support in vivo E-AB sensing. Future research will focus around testing the long-term stability of the probe structure and the E-AB sensor surface. Recent work implicates multiple pathways for the degradation of nucleic acid-based sensors in living systems, including biofouling by macromolecules and competitive displacement of monolayer elements by naturally occurring thiol compounds.^27,28^ A possible remedy to these issues is biocompatible coatings overlaid on top of the sensor surface, forming a physical barrier to exclude macromolecules.^29,30^ Additionally, the effects of long-term exposure to bodily fluids on physical parameters of the probe, such as deformation caused by the resin absorbing fluid, must still be investigated. While predicting the biocompatibility of SLA printed prototypes can be challenging due to the proprietary nature of the photopolymer resins, future work will investigate the effects of the implant on the surrounding tissue via histology and toxicology analyses.^31^

Overall, we believe the streamlined platform presented in this study, along with recent approaches published by others,^26,32,33^ should lower the barrier to entry for laboratories seeking to develop and test in vivo E-AB sensors. With further optimization, it may also enable high-resolution studies of drug distribution and elimination kinetics in the subcutaneous compartment, offering temporally dense datasets for detailed pharmacokinetic analysis.
